# *Klebsiella variicola*: an emerging pathogen in humans

**DOI:** 10.1080/22221751.2019.1634981

**Published:** 2019-07-01

**Authors:** Nadia Rodríguez-Medina, Humberto Barrios-Camacho, Josefina Duran-Bedolla, Ulises Garza-Ramos

**Affiliations:** Instituto Nacional de Salud Pública (INSP), Centro de Investigación Sobre Enfermedades Infecciosas (CISEI), Laboratorio de Resistencia Bacteriana, Cuernavaca, México

**Keywords:** Nitrogen fixation, misclassification, bacterial infections, multidrug-resistant, molecular epidemiology

## Abstract

The *Klebsiella pneumoniae* complex comprises seven *K. pneumoniae-*related species, including *K. variicola*. *K. variicola* is a versatile bacterium capable of colonizing different hosts such as plants, humans, insects and animals. Currently, *K. variicola* is gaining recognition as a cause of several human infections; nevertheless, its virulence profile is not fully characterized. The clinical significance of *K. variicola* infection is hidden by imprecise detection methods that underestimate its real prevalence; however, several methods have been developed to correctly identify this species. Recent studies of carbapenemase-producing and colistin-resistant strains demonstrate a potential reservoir of multidrug-resistant genes. This finding presents an imminent scenario for spreading antimicrobial resistant genes among close relatives and, more concerningly, in clinical and environmental settings. Since *K. variicola* was identified as a novel bacterial species, different research groups have contributed findings elucidating this pathogen; however, important details about its epidemiology, pathogenesis and ecology are still missing. This review highlights the most significant aspects of *K. variicola*, discussing its different phenotypes, mechanisms of resistance, and virulence traits, as well as the types of infections associated with this pathogen.

## Introduction

*The Klebsiella pneumoniae* complex is a member of the *Klebsiella* genus of the family *Enterobacteriaceae*. Recently, the taxonomy of the *K. pneumoniae* complex has expanded. It comprises *K. pneumoniae, K. quasipneumoniae* subsp. *quasipneumoniae*, *K. quasipneumoniae* subsp*. similipneumoniae*, *K. variicola* subsp. *variicola* (for practical purposes, we refer to this subspecies as *K. variicola*)*, K. variicola* subsp. *tropicalensis* and *K. africanensis* bacterial species. *K. quasivariicola* is also included, but remains to be validly published [1,2]. All the members of this complex possess overlapping biochemical and phenotypic features; thus, classifying them by traditional microbiological methods is not practicable [3–5]. Usually, all taxa of the *K*. *pneumoniae* complex are misassigned as *K. pneumoniae,* the major species within the complex. Different alternatives have been proposed to solve this inappropriate identification. The first alternatives developed were based on PCR [6,7]; however, the phylogenetic analysis of housekeeping genes has been routinely used and is highly accurate [5,8,9]. Currently, MALDI-TOF MS (with a properly updated database) and gene and genome sequencing are feasible means of differentiating the bacterial species that constitute the *K*. *pneumoniae* complex [2,10,11].

*K. variicola* is a Gram-negative, facultative anaerobic, and nonmotile bacillus. It forms circular, convex and mucoid colonies and grows at approximately 11–41°C [12]. Infections caused by *K. variicola* have been reported in humans worldwide [6,8,11,13–15] and less frequently in wild and farm animal infections [16,17]. Additionally, *K. variicola* is frequently isolated from a wide range of plant ecosystems, playing a role in nitrogen fixation and plant growth promotion [12,18–23]*.* The inaccurate identification of the members of the *K. pneumoniae* complex has limited the study of *K. variicola,* leaving gaps in knowledge and clinical implications within health care systems. Therefore, the aim of this review is to discuss general aspects of *K. variicola* and highlight the most significant aspects of *K. variicola* as an emerging human pathogen with increasing antimicrobial resistance gene and virulence profiles.

## Description of *Klebsiella variicola* as a novel bacterial species

Brisse et al. (2001) identified genetic diversity within *K. pneumoniae* clinical isolates*.* They assessed these isolates through a phylogenetic analysis of the *parC* and *gyrA* genes [4]. The analysis revealed the existence of three distinct *K. pneumoniae* phylogroups named KpI, KpII and KpIII, with KpI being the most prevalent. In 2004, based on genetic and biochemical assays, *K. variicola* was proposed as a novel bacterial species recovered from plants and clinical isolates that was initially identified as *K. pneumoniae*. Likewise, it was observed that the phylogroup KpIII corresponded to *K. variicola* [5]. The isolates identified as *K. pneumoniae* (using standard biochemical techniques) were subjected to various tests, such as adonitol fermentation, nitrogen fixation through acetylene reduction, phylogenetic analysis of housekeeping genes and DNA–DNA hybridization. The data obtained demonstrated the existence of a new bacterial species named “*variicola*”, which comes from the Latin word va.ri.i’co.la., meaning “inhabitant of different places”[5]. Later, through phylogenetic analysis, Brisse et al. (2014) described that the phylogroup KpII corresponds to *K. quasipneumoniae.* Phylogenomic differences of these phylogroups that support them as distinct and separately evolving population species were subsequently confirmed [3].

The phenotypic and biochemical characteristics of the *K. pneumoniae* complex are extremely similar under standard microbiological conditions [12]. Like all members of the *K. pneumoniae* complex*, K. variicola* is positive for urease, ortho-nitrophenyl-β-galactoside (ONPG), the Voges-Proskauer test, and lysine decarboxylase but negative for indole and ornithine decarboxylase. Unlike the rest of the *K. pneumoniae* complex, both subspecies of *K. variicola* (*K*. *variicola* subsp. *variicola* and *K*. *variicola* subsp. *tropicalensis*) metabolize tricarballylic acid, L-galactonic acid-γ-lactone, L-sorbose, 5-keto-D-gluconic acid, 4-hydroxyl-L-proline and D-arabitol; likewise, the inability to ferment N-acetyl-neuraminic acid is also a particular trait of both subspecies. Moreover, some characteristics that differentiate *K*. *variicola* subsp. *variicola* from *K*. *variicola* subsp. *tropicalensis* are the ability to metabolize mono-methyl succinate, D-lactic acid methyl ester and 3-O-(ß-D-galactopyranosyl)-D-arabinose and the inability to metabolize D-psicose [24,25]. The assimilation of 5-keto-D-gluconate is supported by the presence of the *idnO* gene exclusively in both subspecies of *K. variicola,* and as expected, this gene is absent in *K. pneumoniae* and *K. quasipneumoniae* species [26]. Great efforts have been made to gain a deeper understanding of the biochemical characteristics of the *K. pneumoniae* complex. This information may contribute to a more accurate differentiation of *K. variicola*.

## *K. variicola* misidentification: a problem being resolved?

**Figure 1.**
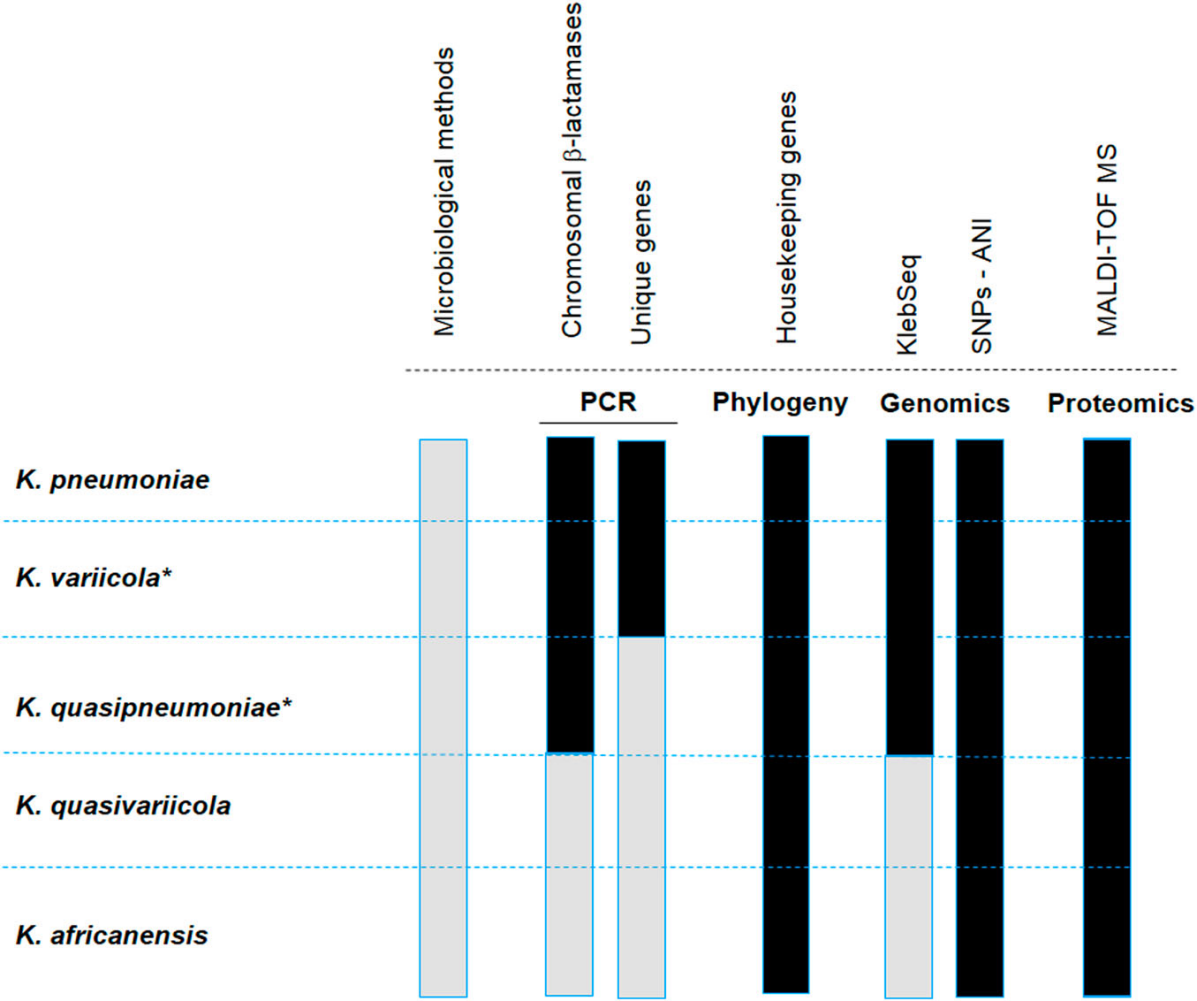
Representation of the available methods that allow differentiating the *K. pneumoniae* complex*.* The black colour means that the indicated method distinguishes the species of the *K. pneumoniae* complex, and the grey colour implies the opposite. Microbiological methods refer to standard biochemical procedures. PCR refers to molecular methods based on the amplification of chromosomal β-lactamases or unique genes assembled in a multiplex PCR, or single PCR such as *yggE*. The phylogenetic analysis corresponds to the most commonly used gene, *rpoB*. KlebSeq is a sequencing tool for screening and epidemiological surveillance of the *Klebsiella* genus. Single nucleotide polymorphisms (SNPs) have been used for the differentiation of some species of the *K. pneumoniae* complex, but they are definitely feasible for all species of the complex. The average nucleotide identity (ANI) is a genomic tool used for species differentiation and is highly recommended for this purpose. With the recent update of the proteomic profile of the *K. pneumoniae* complex, it is possible to differentiate the species that constitute the *K. pneumoniae* complex by using MALDI-TOF*.* The Bruker reference library version 6.0.0.0 includes the reference spectra for proper identification of *K. variicola* from *K. pneumoniae* but not for the rest of the members *of the K. pneumoniae* complex*.* The asterisk symbol behind the species name means that the subspecies are included, for instance *K. variicola* subsp. *variicola, K. variicola* subsp. *tropicalensis*, *K*. *quasipneumoniae* subsp. *quasipneumoniae* and *K*. *quasipneumoniae* subsp. *similipneumoniae*.

The identification of *K. variicola* is not routinely sought in clinical microbiology laboratories, and the misidentification of *K. variicola* as *K. pneumoniae* was documented several years ago [27,28]. Currently, the identification of new bacterial species and subspecies within the *K. pneumoniae* complex, such as *K. quasipneumoniae, K. variicola* subs *tropicalensis, K. africanensis* and *K. quasivariicola,* requires updating the developed protocols to increase the accuracy of identifying new species in the *K. pneumoniae* complex. Fortunately, molecular, genomic and proteomic methods are now available (Figure 1). With the use of these different tools, it will be easier to better differentiate the species within the *K. pneumoniae* complex. An accurate identification of clinical and environmental isolates will help to understand its epidemiology. The available methods are described below.

### Microbiological methods

Automated systems such as Vitek 2XL, Phoenix, and MicroScan are widely used in microbiology and clinical laboratories for the identification of both Gram-negative and Gram-positive bacteria. These microbiological methods are based on colorimetric changes in miniaturized substrates. Because automated systems rely on the general biochemical characteristics of the *K. pneumoniae* complex, it is not possible to differentiate *K. variicola* or any species of the complex. Thus, automated systems will identify all isolates as *K. pneumoniae* (Figure 1).

### Molecular methods: PCR-based on chromosomal β-lactamase genes

Previous analyses have revealed that the chromosomal presence of class A β-lactamase is a distinguishing characteristic among bacteria in the *K. pneumoniae* complex [20,29]. A multiplex PCR system based on the amplification of chromosomal β-lactamase was proposed to identify *K. pneumoniae (bla*_SHV_)*, K. quasipneumoniae* (*bla*_OKP_) and *K. variicola (bla*_LEN_) [30] (Figure 1). This multiplex PCR system amplifies the *bla*_genes_ along with their flanking region (*deoR*) and a gene coding for an ATPase, which is a conserved region within the chromosome. Although the authors included *E. coli* strains with and without a plasmid-borne *bla*_SHV_ as controls, clinical isolates of *K. variicola* with plasmid-borne *bla*_SHV_ have been reported [7,11,31]. Therefore, cross-reactivity with another member of the *K. pneumoniae* complex that encodes mainly the broadly disseminated plasmid-borne SHV β-lactamase family should be considered. In addition, the fact that *K. variicola* harboured the OKP β-lactamase instead of the LEN β-lactamase makes it difficult to identify the members of the *K. pneumoniae* complex when applying gene-based systems based on β-lactamase proteins [11].

#### PCR-based on unique genes

Yasuhara-Bell et al*.* (2015) designed a diagnostic molecular tool based on loop-mediated isothermal amplification (LAMP) for detecting *K. variicola* and *K. oxytoca* isolates from infected ironwood trees (Figure 1). This assay was accomplished by whole-genome comparisons to obtain specific proteins for both bacterial species. Two proteins were selected: i) hemolysin-coregulated protein 1 (Hcp1) unique to *K. variicola* and ii) UGH, a glucuronyl hydrolase unique to *K. oxytoca* [32]. Practically simultaneously, Garza-Ramos et al. (2015) [7] and Berry et al. (2015) [6] proposed the first efficient molecular methods for proper differentiation of *K. variicola* from *K. pneumoniae*. Berry et al. (2015) described a case of a bloodstream infection (BSI) caused by a “not detected” bacterium when using the Verigene system. The isolate was identified as *K. pneumoniae* through the Vitek 2XL system and was subsequently identified as *K. variicola* by phylogenetic analysis of the *yggE* gene [6] (Figure 1). Later, a collection of *K. variicola* isolates was identified by a *yggE* PCR-RFLP assay and MALDI-TOF MS. Since *yggE* PCR-RFLP had 94.6% coincidence with MALDI-TOF results, this finding suggests that the *yggE* gene may be suitable for *K. variicola* identification [14]. Garza-Ramos et al. (2015) developed a multiplex PCR probe system based on comparative genomics of *K. variicola* and *K. pneumoniae* genomes to identify shared unique proteins of each bacterial species (Figure 1). This multiplex PCR (M-PCR-1) consists of the amplification of three different amplicons: a phosphohydrolase (888 bp) for *K. pneumoniae*, a phosphoglycerate mutase (449 bp) for *K. variicola,* and the *mtnC* gene (340 bp) used as a molecular marker for both species. This probe system was used for screening isolates of *K*. *variicola* among 1,060 *K. pneumoniae* clinical isolates obtained from several Mexican hospitals. The authors found that *K. variicola* had a prevalence of 2.1%. Half of the isolates identified corresponded to multidrug-resistant isolates, which were determined to be extended-spectrum β-lactamase (ESBL)-producers. All *K. variicola* isolates were validated by phylogenetic analysis of *rpoB* genes, regarding which we consider that the multiplex-PCR probe system could identify *K. variicola* with high accuracy [30]. This multiplex PCR (M-PCR-1) has been recently been used in screenings allowing the identification of *K. variicola* isolates in plants as well as in hospital- and community-acquired infections [13,23,33,34]. Even a *K. quasipneumoniae* clinical isolate (with a hypermucoviscous phenotype) could be indirectly determined because when an isolate is determined biochemically as *K. pneumoniae* (MicroScan Walkaway system in this case) but negative to probes used in the M-PCR-1, later it was confirmed that the clinical isolate corresponded to *K. quasipneumoniae* [35].

The molecular methods are based on phylogenetic analysis of 16S rRNA and *rpoB* genes, with this last gene being the most widely used to properly identify isolates of *K. variicola* [8,36] (Figure 1). Likewise, the use of the *rpoB* gene has been reported to be more accurate at *Klebsiella* species identification [37]. Some strains of *K. variicola* have been identified from several sources through *rpoB* phylogenetic analysis, including animals such as dogs, birds, monkeys, and cows (mastitis) and plants such as coriander (food supply) [16,17,38]. Moreover, a clinical strain of *K. variicola* causing fatal sepsis was initially misidentified by the MicroScan platform and later identified correctly through *rpoB* phylogenetic analysis [15]. The phylogenetic analysis of concatenate housekeeping genes has been proposed [3,7], although it has not been tested with the new species of the *K. pneumoniae* complex.

### Genomic-based and automated methods

The development of genomics has grown with next-generation sequencing platforms allowing the whole-genome sequencing (WGS) of bacterial pathogens. WGS has gained applications in health care diagnostics for screening and surveillance of bacterial pathogens, outbreak detection, and source tracing of major pathogens [39]. Although the WGS approach is not currently cost-effective worldwide, other sequencing alternatives have been proposed not only for detecting but also for characterizing pathogens. The KlebSeq sequencing tool is a promising tool for screening and epidemiological surveillance of the *Klebsiella* genus*,* which includes the identification of some members of the *K. pneumoniae* complex (*K. pneumoniae*, *K. variicola* and *K. quasipneumoniae)* without a culturing step [10] (Figure 1). Hence, the species identification by means of KlebSeq is based on sequencing of target genes. In the case of *K. variicola* and *K. quasipneumoniae*, KlebSeq uses single nucleotide polymorphism (SNP) amplicon analysis for a specific gene. Although KlebSeq was designed for characterizing *K. pneumoniae* isolates, updating this platform to include the recently described *Klebsiella* species and information such as capsule type, antibiotic resistance and virulence genes for all the members that constitute the *K. pneumoniae* complex would contribute to understanding the epidemiological background of these species.

The use of SNP-base analysis for *Klebsiella* species identification was used previously by Holt et al. (2915) [1]. They identified 175,120 SNPs within the core genome of 328 *Klebsiella* genomes with these SNPs a phylogenetic analysis revealed a split-network three clearly separated groups corresponding to *K. pneumoniae*, *K. variicola* and *K. quasipneumoniae*. Another option is the use of average nucleotide identity (ANI), a powerful tool for resolving genomic relatedness between strains (Figure 1). ANI is calculated from pairwise comparisons of all sequences shared between two genomes [40]. While ANI is not a *K. variicola*-specific genomic tool, several studies have reported its utility in resolving species differentiation. Clearly, misidentification at the microbiological level drives an incorrect genome classification, and several examples of misclassification have been described [9,26]. The WGS approaches are useful in pathogen and outbreak detection [39]. There are several examples involving bacterial pathogens; however, for the *Klebsiella pneumoniae* complex, a clear example is the epidemiological study of 1,777 sequenced genomes; this approach allowed the identification of both *K. variicola* and *K. quasipneumoniae* ESBL- and carbapenemase-producing isolates [11].

MALDI-TOF MS is a fast and cost-effective technique [41,42], while the Bruker microorganism reference library version 4.0.0.0 does not include a *K. variicola* profile [11]. Moreover, the Bruker library version 6.0.0.0 includes reference spectra for *K. variicola* and *K. pneumoniae.* Recently specific biomarkers for all bacterial species of the *K. pneumoniae* complex were reported using MALDI-TOF MS [2] (Figure 1). The authors found different combinations of peaks in the spectra that highly correlate with the *K. pneumoniae* complex, mainly corresponding to ribosomal proteins. In the case of *K. variicola,* its identification is based on the L31 ribosomal protein. Thus, there is opportunity for updating MALDI-TOF databases to correctly differentiate the bacterial species of the *K. pneumoniae* complex and exploit the potential of MALDI-TOF platforms for discriminating this species [43].

Several methods are available for correctly differentiating *K. variicola* within the *K. pneumoniae* complex; however, the best approaches are MALDI-TOF MS (with a properly updated database), PCR systems based on single or multiple genes (*yggE* or multiplex PCR probes) and ANI using the sequenced genome. Unless these methods are routinely adopted in clinical microbiology laboratories, misidentification will continue to be a problem.

## Environmental distribution of *K. variicola*

In addition to the clinical environment where *K. variicola* has been identified, this bacterial species is also found in a wide diversity of natural niches. The mutualistic relationship between leaf-cutter ant colonies and fungus was the first example. The successful colony growth of leaf-cutter ants is supported by the symbiotic nitrogen fixation of *K. variicola*, suggesting that colonies obtain a substantial proportion of their nitrogen requirements from this bacterial association [22]. Likewise, the misidentified *K. variicola* KP5-1 strain was isolated from the microbiota of wild southern green stink bugs (*Nezara viridula*). The potential of this insect as a vector of cotton disease-causing agents is well known. The opportunistic *K. variicola* has been documented to cause appreciable boll damage [44]. In China, *K. variicola* KV321 was isolated from rhizosphere soil of *Pisolithus tinctorius-Eucalyptus* mycorrhiza. The isolate exhibited high nitrogen-ﬁxation ability, and the antibiotic susceptibility tests demonstrated resistance to polymyxin, penicillin G, tetracycline, and erythromycin [45]. Nitrogen cycling and diversity of the organisms that carry out nitrogen transformations in subterranean habitats is poorly studied. Some of the samples of bacterial mats collected from lava cave walls were identified as nitrogen-fixating *K. pneumoniae* by the presence of *nifH* genes. Analysis of reported *nifH* sequences revealed a possible misidentification of these strains, and the results revealed a *K. variicola*-like subset of the nitrogen-fixating bacterial community in the subterranean habitats [46].

The potential capacities of *K. variicola* to be involved in or produce diseases are not limited only to wildlife animals. Bovine mastitis is the condition where the animal displays physical symptoms in the udder, such as swelling, heat, hardness, redness, pain and milk exhibiting a watery appearance, flakes, clots, or pus. The diseased cow presents a decrease in milk yield that depends on the specific pathogen causing the infection, where *K. pneumoniae* is one of the primarily microbial agents responsible for environment-derived mastitis. In Newfoundland, Canada, 4% of bovine mastitis cases from 11 farms were identified as being caused by *K. variicola*. The pathogenicity of *K. variicola* as an infectious agent in bovines is not well known [17]. Diseases produced by *K. variicola* are widely extended to different organisms, such as ironwood (*Casuarina equisetifolia* subsp. *equisetifolia*), a nitrogen-fixing tree of considerable social, economic and environmental importance in zones of Asia, the Pacific, Africa, and Central America. The decline in ironwood was first reported in Guam in 2002 and associated with wetwood symptoms and is now affecting thousands of trees and the ecosystem. Bacterial samples obtained from diseased ironwood trees were identified as containing the common pathogen *Ralstonia solanacearum* associated with *K. variicola* and *Klebsiella oxytoca*. Causative agents of wetwood in the ironwood tree decline worldwide have not been completely determined, but scientists attribute the disease to a complex association between *R. solanacearum*, *K variicola* and *K.* oxytoca [47].

Likewise, *K. variicola* can be found in several associations with its environment, such as contaminated soil, rivers, and wastewater [19,36,48,49]. Due to its potential for use in industrial processes, *K. variicola* has been used for the development of biotechnological and environmental protections, such as a source of renewable energy, fermentation and energy biocatalysts, waste degradation, wastewater treatment, bioremediation of pollutants and biodegradation [19,36,48,50,51].

There is clear evidence that plants are a natural reservoir of *K. variicola,* and in some cases, this niche is shared with *K. pneumoniae* [23]; however, this aspect is poorly studied. Recently, the term “phytonosis” was proposed in order to describe human infections caused by plant-borne bacteria, parallel to the term zoonosis for animal-borne bacterial pathogens [20]. Later, phytonosis was described more as a case of kingdom-crossing bacteria [52]. This term was used for the *K. variicola* X39 clinical isolate, which had the capacity to colonize maize roots, stems and leaves in a few days. This dual capacity (human infections and plant colonization) of *K. variicola* was determined previously by *in vitro* assays of plant growth promoting mechanisms, which were shared among plant and human isolates [20]. Today, *K. variicola* can be considered a bacterial species that can infect humans as a pathogen and colonize plants as an endophyte and in few cases as pathogen.

## Emergence of *K. variicola* as a human pathogen

Historically, *K. pneumoniae* has been the underlying cause of a wide range of healthcare-associated infections (HCaIs) and community-acquired infections (CAIs), with an increase in morbidity and mortality [11,53,54]. *K. variicola,* as well as *K. pneumoniae,* is an opportunistic pathogen responsible for infections such as BSIs, respiratory tract infections and urinary tract infections (UTIs) [1,8,11,14,31]. As previously mentioned, most of the cases in which *K. variicola* was the cause of the infection were first identified by biochemical testing as being caused by *K. pneumoniae* [6,11,13,15,29,31,33].

BSIs represent a major challenge in health care systems, and timely pathogen detection is crucial for effective antibiotic therapy [55]. Among the members of the *K. pneumoniae* complex, *K. pneumoniae* is the leading cause of BSI; however, patients diagnosed with a BSI caused by *K. variicola* have a high mortality rate [8]. The first case of *K. variicola* BSI occurred as part of a pediatric outbreak. The BSI was caused by multidrug-resistant and ESBL-producing *K. variicola* clones; however, the clinical outcome was unknown [7,31]. Later, a report of 139 BSIs in a 30-day mortality retrospective study was described. *K. variicola* had the highest mortality rate (29.4%, 10/34 cases), in contrast with a 13.5% (13/96 cases) mortality rate associated with *K. pneumoniae* infections. Intriguingly, these isolates do not contain additional known virulence factors that could explain the mortality. In this study, *K. variicola* was isolated more frequently (24.4%, 34/139) than in other studies [8]. In addition, several other reports have clearly demonstrated that *K. variicola* is associated with BSI [6,17,56,57]. Recently, an outbreak of neonatal sepsis caused by *K. variicola* was described by Farzana et al. (2018). They undertook an integral study of isolates causing sepsis in neonates and found a significant mortality attributable to *K. variicola.* This clone possessed virulence genes typically found in any other *K. pneumoniae* strain and, most importantly, the acquired antimicrobial resistance genes NDM-1 and CTX-M-15 [56].

*K. variicola* has also been associated with infections in immunocompromised individuals. Some comorbidities, such as systemic lupus erythematous, cancer, diabetes mellitus, hepatobiliary diseases, solid organ transplantation and alcoholism, have been reported in several studies [6,15,58,59]. Berry et al. (2015) described a case of BSI caused by *K. variicola* in a female patient with systematic lupus erythematosus. In Japan, fatal sepsis was attributable to *K. variicola* (originally misidentified as *K. pneumoniae).* The patient died despite the immediate administration of antibiotics to which *K. variicola* was susceptible [15]. Another report in which *K. variicola* was isolated from blood samples was described by Ledeboer et al. (2015). They reported that 25/26 blood cultures first reported as *K. pneumoniae* were subsequently identified as *K. variicola*. These isolates were collected in different clinical centres to validate a diagnostic tool specific for Gram-negative bacteria causing BSI [6,57].

*K. variicola* and *K. pneumoniae* can infect the same patient [13]. In Mexico, an interesting case occurred in which a patient was infected by susceptible and carbapenem-producing *K. pneumoniae* at different time intervals. The susceptible isolates were recovered from a BSI and subsequently identified both molecularly and phylogenetically as *K. variicola*. Both isolates had different phenotypes and genotypes observed by pulsed-field gel electrophoresis (PFGE) analysis and displayed different plasmid profiles.

UTIs represent an important public health problem that is accompanied by a significant economic burden. UTIs are primarily caused by Gram-negative bacteria; *K*. *pneumoniae* is the second-most prevalent pathogen causing UTIs [60]. Given the misidentification problem, *K. variicola* is an underrecognized pathogen capable of causing UTIs. Recently, a retrospective study carried out in a collection of *K. variicola* clinical isolates illustrated that 70% (39/56) of the *K. variicola* isolates were recovered from UTIs. From these data, the authors demonstrated the potential of some strains to cause UTIs using a murine model. In addition, the uropathogenicity of *K. variicola* was associated with novel usher fimbrial proteins and differential expression of type 1 fimbrial proteins [14].

In health care systems, *K. variicola* produces a minor number of infections compared to those of *K. pneumoniae*; however, infections caused by *K. variicola* could be as severe as *K pneumoniae* infections [8,11]. Usually, *K. variicola* isolates display lower antibiotic resistance rates than *K. pneumoniae* [7,13]; however, this fact is not necessarily associated with a better treatment response [6,15].

## Emergence of multidrug-resistant strains

**Figure 2.**
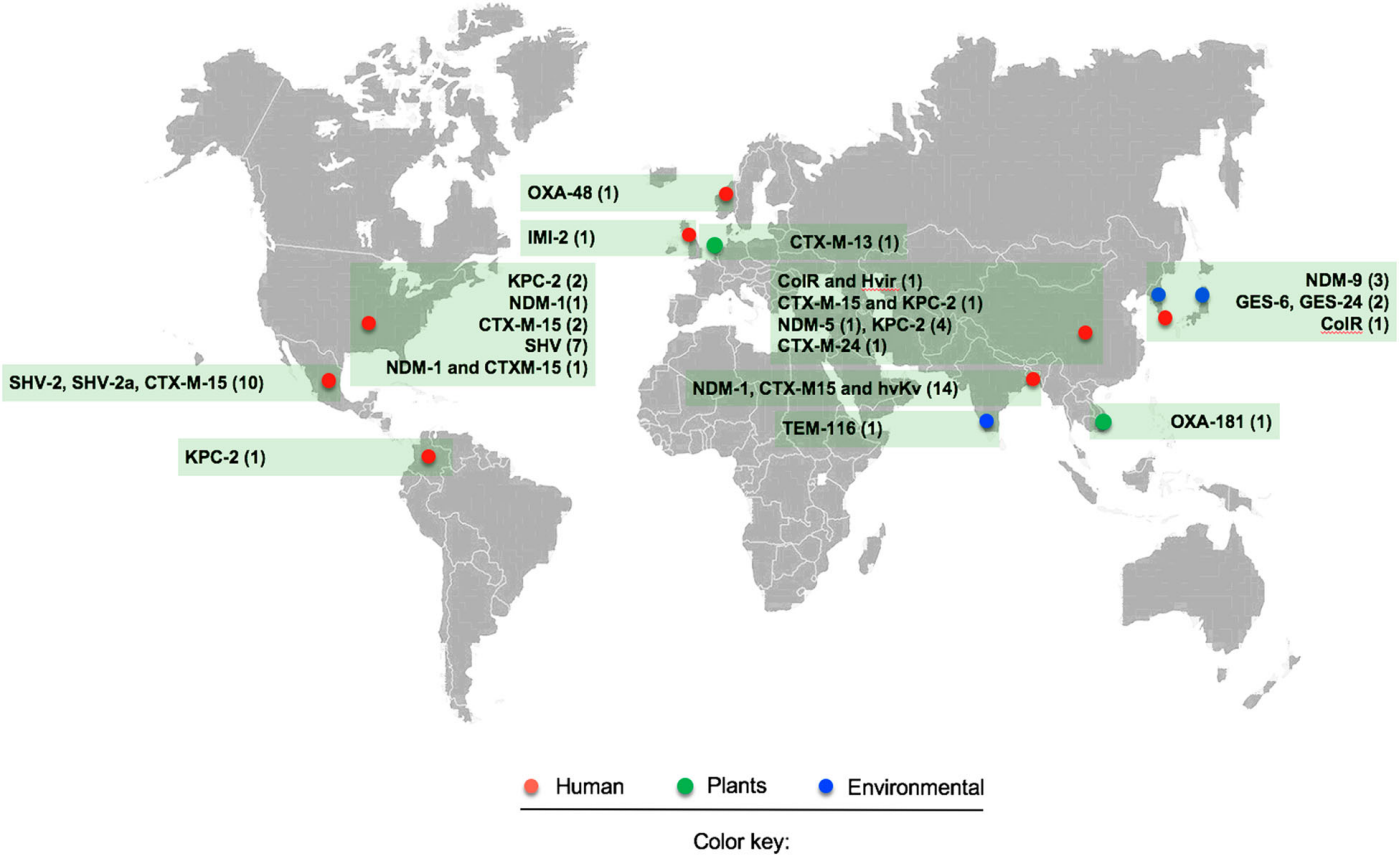
Map of reported multidrug-resistant (MDR) and hypervirulent *K. variicola* (hvKv) isolates from different isolation sources. The red circle indicates clinical isolates, the green circle refers to *K. variicola* plant isolates and the blue circle represents environmental isolates. The numerals between parentheses indicate the number of isolates reported for both MDR and hvKv.

*K. variicola* is intrinsically resistant to ampicillin due to the presence of the chromosomal β-lactamase LEN; however, it is susceptible to most antibiotic classes [7,11,14,20,29]. This pattern has changed over time due to an increase in the number of reports of multidrug-resistant *K. variicola* isolates (Figure 2) [7,10,14,31,61–66]. Likewise, reports of ESBL- and carbapenemase-producing *K. variicola* isolates have been increasing even though some strains were not isolated from clinical settings, which highlights a key role of the environment as reservoirs of antimicrobial resistance genes (Figure 2) [38,67].

The first case reported in a clinical setting goes back to a pediatric hospital outbreak that occurred in 1996 in Mexico. The isolates were positive for ESBL of the SHV-type, for which the β-lactamase gene was located on a conjugative plasmid [7,31]. Afterward, an IMI-2 carbapenemase-producing *K. variicola* strain was obtained from a soft tissue infection of a patient in the intensive therapy unit in the UK. The strain was resistant to ertapenem, meropenem and imipenem (MIC ≥32 mg/L) but susceptible to cefotaxime and ceftazidime (MIC ≥0.25 mg/L). The IMI-2 carbapenemase was located on a transferable plasmid with a size of 77,843 bp and from the IncFII_γ_ family; however, this plasmid did not encode any other known resistance genes. *In silico* analysis revealed high similarities with other published IncFII_γ_ plasmids, of which many, such as the NDM-, IMI-, IMP- or KPC-type families, also harboured a carbapenemase gene (Figure 2) [62].

More recently, Long et al. (2017) demonstrated that *K. pneumoniae*, *K quasipneumoniae* and *K*. *variicola* share chromosomal and mobile genes, which encode virulence factors and antimicrobial resistance genes. In this study, 15/1,777 and 13/1,777 of clinical isolates were identified as *K. quasipneumoniae* and *K. variicola,* respectively. *K. variicola* isolates were identified as ESBL-producing (9/13) or carbapenemase-producing (3/13) isolates. The ESBLs identified were SHV-type in 7/13 (SHV-5, −12, -30 alleles) isolates and CTX-M-15 in 2/13 isolates. The carbapenemases identified were KPC-2 in 2/13 isolates and NDM-1 in 1/13 isolates. One *K. variicola* isolate contained both the CTX-M-15 and NDM-1 genes [11]. A recent report of genome sequence-based analysis of carbapenemase-producing isolates among the *K. pneumoniae* complex was described [64]. The authors reported the presence of KPC-2 (4/5) and NDM-5 (1/5) in clinical isolates of *K*. *variicola*; this report is the first description of *K. variicola* producing the NDM-5 allele. The analysis of the distribution of antimicrobial resistant genes by means of genomic approaches has revealed the presence of an OXA-48-producing isolate recovered from a hospitalized patient in Norway (Figure 2) [68].

Colistin resistance mediated by chromosomal mechanisms in *K. variicola* was recently described [63]. Joo E et al. (2018) analyzed fecal samples from healthy Korean adults to evaluate fecal carriage of antibiotic-resistant pathogenic *Enterobacteriaceae.* A total of 1,417 fecal samples were analyzed, 4.5% (64/1417) from which Gram-negative isolates were obtained, and chief among them (14%, 9/64) were *Klebsiella* spp. The distribution of species within these isolates was 44.4% (4/9) identified as *K. pneumoniae*, 33.3% (3/9) identified as *K. oxytoca* and 22.2% (2/9) identified as *K. variicola*. One *K. variicola* isolate displayed colistin resistance (MIC 32 mg/L) without the presence of the *mcr-1* gene, and because of this result, the authors considered a chromosomal mechanism; however, no further analysis was performed. Later, Lu et al. (2018) described the first report of hypervirulence associated with colistin resistance (see detailed information below) [66]. Colistin resistance was mediated by chromosomal modification of a two-component regulatory system (PhoP-PhoQ). The substitution of D150G in PhoP, which is known to mediate colistin resistance, was found (Figure 2) [69].

With respect to environmental settings, Zurfluh et al. (2015) reported the first detection of *K. variicola* harbouring OXA-181 carbapenemase in fresh vegetables (coriander) imported from Asia to Switzerland [38]. The OXA-181 carbapenemase gene was encoded on an IncX3-type plasmid of 51,480 bp and flanked by two IS26-like elements without the capacity of transference. In addition, the emergence of *K. variicola* producing NDM-9 was described in a South Korean urban river; the NDM-9 gene was located on a 108-kb IncFIII(Y) plasmid, and a mercury resistance operon was located upstream of NDM-9 flanked by IS26-TnAS3 and IS15, IS26 and IS15D1 [67]. Moreover, the presence of GES-6 and GES-24 in wastewater samples has also been reported [70]. Interestingly, the genetic context of these carbapenemase genes was found in two distinct integrons, *In1439,* which contains the array *bla*_GES-24_-*aacA4,* and *In1442,* which contains *bla*_GES-6_-*aacA4-bla*_OXA-17_ (Figure 2). These data highlight the existence of possible routes for the dissemination of carbapenemase-producing *K. variicola* in the environment through food and water [38]. Dissemination of carbapenemases is accompanied by other antibiotic resistance genes within highly transferable mobile genetic elements that enhance the possibility of propagation among microorganisms in the environment.

Plasmids play an important role in the dissemination of antimicrobial resistance and virulence genes [71]. *K. variicola* shares a similar distribution of plasmids with the other members of the *K. pneumoniae* complex, suggesting that horizontal gene transfers between these members occur and favour the spread of both antimicrobial resistance and virulence genes. Particularly, the FIBk, FIIk, and FII replicon types were found mostly in both *K. variicola* and *K. quasipneumoniae* isolates [11].

The genomic studies described above have contributed to understanding the distribution of antimicrobial resistant genes in *K. variicola*. The recent acquisition of antimicrobial resistance genes highlights the possibility of horizontal gene transfer as the main mechanism in the adaptation of *K. variicola*. These studies demonstrate the importance of precise identification since this bacterial species is becoming a concern because of its potential to acquire and disseminate antimicrobial resistance genes in clinical and environmental settings. The map presented in Figure 2 is likely an underestimate of resistant *K. variicola* infections, as the majority are still misclassified in clinical laboratories.

## Virulence and pathogenicity traits

Most of the virulence factors displayed in *K. variicola* correspond to factors described previously in *K. pneumoniae. K. variicola* exhibits four major virulence factors: capsule, lipopolysaccharide (LPS), siderophores and fimbriae. It has been suggested that *K*. *variicola* can be as virulent as *K*. *pneumoniae* strains [8,11]. The first study that analyzed the virulence factors and determined the pathogenicity of *K. variicola* was performed in a single strain (*K. variicola* 342) obtained from maize [18]. This isolate was initially misidentified as *K. pneumoniae* [7,26]. The authors proved that *K. variicola* 342 was able to produce UTIs and lung infections in an animal model. Likewise, the authors examined the presence or absence of virulence factors identified in previous studies by signature-tagged mutagenesis or IVET approaches using *K. pneumoniae* strains. They found the genes *wecA*, *msrA*, and *orf*KPK-3791 and a type IV transfer system to be present in the *K. variicola* 342 genome but absent from the multidrug-resistant strain *K. pneumoniae* MGH78578. Nonetheless, these genes were not fully characterized [18].

Screening of virulence-associated genes was carried out in sets of *K. variicola* genomes in independent works [14,20,72]. As expected, the virulence genes that were ubiquitous among *K. variicola* were those involved in type 3 and 1 fimbriae biosynthesis (*mrk, fim*), metabolism of urea (*ureA),* biosynthesis of the core lipopolysaccharide (*wabG*) and iron uptake systems, primarily the enterobactin (*entB*) and *KfuABC* iron systems. The presence of the aerobactin siderophore receptor (*iutA*) was distributed among all *K. variicola* genomes, but no genes encoding the proteins responsible for the biosynthesis of the siderophore itself were found (*iucABCD* cluster) [20]. Other virulence genes found in a minority of genomes were glycerate pathway genes, allantoin utilization genes and the iron uptake systems yersiniabactin (*ybtAESTX* cluster) and salmochelin (*iroN*) [14,20]. The main virulence genes found in *K. variicola* are chromosomally located; however, hypervirulent strains of *K. variicola* harbour virulence genes in large plasmids, such as the siderophores aerobactin, salmochelin, and yersiniabactin and the regulators of the mucoid phenotype *rmpA* and *rmpA2* (see detailed information below) [66].

Interestingly, recent findings about *K. variicola* uropathogenicity have demonstrated significant genetic variability in the type 1 fimbriae system that changes its expression, and nine novel fimbrial usher proteins (Kva and Kvi families) distributed among distinct *K. variicola* clades are involved in their role in uropathogenicity [14]. Martinez-Romero et al. (2017) [20] also described a new fimbrial protein (FimV) found in the set of unique proteins of the *K. variicola* pangenome. FimV was distributed among *K. variicola* genomes except for the BIDMC90, KTE92 and B1 genomes. Its role in pathogenicity is unknown.

*K. variicola* has a heterogeneous distribution of canonical *K. pneumoniae* virulence-associated genes, but in most cases, they are rarely found in *K. variicola* except for those classical virulence factors (capsule, adhesins and siderophores). *K. variicola* possesses its own virulome, which is not completely determined, as is the case for the UTI-associated virulence genes that were elucidated [14]. However, it is unclear whether specific virulence mechanisms are linked to severe infections, mainly BSIs, which are the most common infections produced by *K. variicola.* It is likely that there are other novel virulence genes responsible for this type of infection [8,56]. Like that of *K. pneumoniae* and *K. quasipneumoniae,* the genome of *K. variicola* is considered an open genome, which implies that the organism has the capacity to continue incorporating genes that allow it to adapt to different environments by conferring resistance to antibiotics to which it previously was susceptible, as well as expanding its pathogenicity by incorporating virulence factors [1].

## Classical, hypermucoviscous and hypervirulent strains

Currently, there are two well-recognized *K. pneumoniae* phenotypes: classic (cKpn) and hypervirulent (hvKpn) (Table 1) [73]. cKpn strains occur mainly in hospitals and long-term care facilities. Most classic strains are resistant to a wide range of antibiotic classes, thus clinical management is challenging. Otherwise, hvKpn differs from classical strains due to its capacity to cause infections in healthy and immunocompromised populations in the community [74]. Table 1 summarizes the set of genetic and phenotypic traits that define the cKpn and hvKpn variants. The increased siderophore production (mainly aerobactin (*iucA*) and salmochelin (*iroB*)), capsule overproduction (hypermucoviscous phenotype) driven by *rmpA* and/or *rmpA2* and the presence of new proposed biomarkers such as *peg-344* gene are associated with severe disease or death in murine model assessments [74]. Both *K. pneumoniae* variants are supported by genomic analysis; thus, it is clear that cKpn and hvKpn form two well-separated lineages [75,76]. Moreover, the last few years have seen increasing reports of hypermucoviscous *K. pneumoniae* (hmvKpn) negative for the *rmpA/rmpA2* genes (Table 1)[73,77,78], but these bacteria are poorly characterized, putting into the question the key role of the hypermucoviscous phenotype without a hypervirulence background.

**Table 1.** Genetic and phenotypic traits of *K. variicola* and *K. pneumoniae* distinct phenotypes.

Bacterial species	Phenotype^a^	Parameter									
		Types of infection	Susceptible population	Primary acquired infection type	Virulence factors^b^	Antibiotic resistance phenotype	String test^c^	Capsule (KL)^d^	Sequence type (ST)^e^	AMR genes associated with ST^f^	References
***K. pneumoniae***	**cKpn**	Bacteremia, UTI, pneumonia, meningitis	Immunosuppressed	HAI	Capsule, LPS, fimbriae type 3 and 1, *ybt*, *kfu* (all)	Multidrug-resistant	Negative	KL3 to KL134	ST258 ST512 ST11 ST340 ST437	KPC-2	75,97,98
	**hmvKpn**	Bacteremia and invasive syndromes	Immunosuppressed	HAI	Capsule, LPS, fimbriae type 3 and 1***,****ent* (all)*, ybt*	Susceptible and multidrug resistant	Positive	KL3 to KL134	ST1007 ST1022 and twenty-three different ST	ESBL-producers and quinolone resistance	16,19, 54, 55
	**hvKpn**	Pyogenic liver abscess; bacteremia; lung, neck, and kidney abscesses; pneumonia; cellulitis; necrotizing fasciitis; myositis, meningitis; endophthalmitis	Healthy and immunosuppressed	CAI	*_p_iucA, _p_iroB, peg-344, _p_rmpA,*and *_p_rmpA2*	Mostly susceptible and less frequently multidrug-resistant	Positive	KL1, KL2 and KL54	ST23 ST86 ST375 ST380	KPC-2 CTX-M-3	23,75, 93, 94,98, 99
***K. variicola***	**cKv**	Bacteremia, UTI, pneumonia	Immunosuppressed	HAI and CAI	Capsule, LPS, fimbriae type 3 and 1, *kva*, *kvi*, *fimV*, *ybt*, *ent* (all), *kfu* (all)	Susceptible and multidrug resistant	Negative	KL3 to KL134	ST76 ND ST64 ST92 ST125 ST75	CTX-M-15/NDM-1 OXA-48 NDM-9 CTXM-15/KPC-2 CTXM-14/KPC-2 KPC-2	31, 32, 36, 62, 65, 69, 81
	**hmvKv**	Pneumonia	Immunosuppressed	HAI	Capsule, LPS, fimbriae type 3 and 1, *fimV, ent* (all)*, kfu* (all)	Susceptible	Positive	KL114	ST2	Negative	34
	**hvKv**	Bacteremia	Immunosuppressed	HAI	R1. _p_*iucA*, _p_*iroB*, _p_*ybt*, *ent* (all), _p_*rmpA*, and _p_Δ*rmpA2*	Susceptible to most antibiotic classes except for colistin	Negative	KL16	ND	D150G in PhoP	64
					R2. Capsule, LPS, fimbriae type 3 and 1, *ent* (all), *kfu* (all)	Multidrug-resistant	Negative	Novel KL	ST60	CTX-M-15 NDM-1	26

^a^cKpn, classical *K. pneumoniae*; hmvKpn, hypermucoviscous *K. pneumoniae*; hvKpn, hypervirulent *K. pneumoniae*; cKv, classical *K. variicola*; hmvKv, hypermucoviscous *K. variicola*; hvKv, hypervirulent *K. variicola*.

^b^Underline characters refers to new fimbrial type 1 systems; all, means identified the entire population of isolates analyzed. R1: refers to hypervirulent *K. variicola* colistin resistant and R2: refers to hypervirulent *K. variicola* responsible for causing an outbreak;_p,_ refers to plasmid localization of virulence factor

^c^Positive string test define hypermucoviscous strains.

^d^Capsule type determined by using *K. pneumoniae* capsule typing scheme.

^e^ST was determined by using the MLST *K. pneumoniae* (https://bigsdb.pasteur.fr/klebsiella/klebsiella.html) [79] and MLST *K. variicola* (http://mlstkv.insp.mx) [80] schemes for *K. pneumoniae* and *K. variicola* isolates, respectively. In the case of classic *K. variicola* and *K. pneumoniae* the STs refers to clinically important MDR clones. ND; corresponding to *K. variicola* genomes without ST which was not possible to determine due to fragmentation of one from seven locus from MLST *K. variicola* scheme. Additionally other ST of MDR *K. variicola* with unpublished data could be consulted in Barrios-Camacho *et al*, 2019 [80].

^f^AMR; antimicrobial resistance genes.

Similar to the phenotypes observed in *K. pneumoniae,* three distinct phenotypes may be displayed by *K*. *variicola*: classic (cKv), hypermucoviscous (hmvKv) and hypervirulent (hvKv) (Table 1). This classification is based on reports in the literature rather than genomic traits; however, with the increasing number of *K. variicola* genomes, it would be better to have a deep understanding of the correlation between phenotypic and genomic traits. We defined i) cKv as both multidrug-resistant and susceptible isolates that lacked excessive capsule production (nonhypermucoviscous); ii) hmvKv as one isolate that was positive for the string test (hypermucoviscous) however, unlike hypervirulent *K. pneumoniae,* this isolate was negative for the *rmpA/rmpA2* genes; and iii) hvKv as isolates in which higher or similar mortality rates were observed compared to those of hypervirulent strains of *K. pneumoniae* (Table 1)*.* Intriguingly*,* in both cases, the isolates were reported as hypervirulent, although by different mechanisms (see detailed information below) [43,49,52]. The emergence of these phenotypes in *K. variicola* should be considered when the isolates are being characterized.

### Classic *K. variicola*

*K. variicola* was first identified in plant and clinical settings. As mentioned previously, *K. variicola* isolates are broadly antimicrobial susceptible; however, reports of ESBL-producing and carbapenemase-producing *K. variicola* have increased. In general, both antimicrobial susceptible and multidrug-resistant isolates could be considered classical strains of *K. variicola*. cKva isolates contain virulence factors shared with *K. pneumoniae;* however, cKva has been demonstrated to be pathogenic in BSIs, which could indicate undiscovered virulence factors [7,8,14,54,57,64]. Clinically important multidrug-resistant clones are mentioned in Table 1.

### Hypermucoviscous *K. variicola*

The hypermucoviscous (hmv) phenotype was first described in hypervirulent *K. pneumoniae* (hvKpn). This phenotypic trait is defined by the formation of a viscous filament of ≥5 mm (positive string test) [81]; however, a modified string test has been proposed to avoid discrepancies between clinical laboratories. The tested strain should be inoculated onto agar plates with 5% sheep blood and evaluated for the formation of a filament >10 mm long [82]. The first hmv *K. variicola* isolate was described by Garza-Ramos et al. (2015) [34]. In this report, the authors described the draft genome sequence of the clinical isolate *K. variicola* 8917 obtained from hospitalized elderly patients. Virulence-associated genes, capsule type and the sequence type (ST) were predicted from the genome sequence. Classical virulence factors were found, and among them were siderophores, iron uptake systems, pili and adhesins (Table 1). However, the *rmpA* and *rpmA2* genes associated with the hypermucoviscous phenotype in hvKpn clones were absent. The capsular type was determined by using *wzc* K-typing corresponding to the wzc-932 allele. However, recent findings regarding the genomic diversity of the K-locus suggest that molecular typing based on a single gene, either *wzc* or *wzi,* is not accurate enough; instead, a complete K-locus analysis is suggested to increase the accuracy of K-typing [83]. Considering these results, we analyzed the complete K-locus using the KAPTIVE tool and identified that *K. variicola* 8917 corresponded to the KL114 capsular type. The ST2 was determined using a multilocus sequence typing (MLST) *K. variicola* scheme [80]. *K. variicola* 8917 bears an ∼200-kb plasmid (pKV8917), in which no antibiotic resistance genes were identified. Nevertheless, pKV8917 harbours the tellurium resistance genes *terZABCDE* and *terW* (*ter* operon), which are associated with hvKpn [84]. pKV8917 plasmid curing causes the loss of the hmv phenotype; therefore, pKV8917 harbours the locus responsible for the hmv phenotype in *K. variicola* 8917 (unpublished data). To date, no other reports regarding *K. variicola* with the hmv phenotype have been described.

Other species besides *K. variicola* and *K. pneumoniae* have been described as exhibiting the hmv phenotype: *K. quasipneumoniae* subs. *similipneumoniae* and *K. quasipneumoniae* subsp. *quasipneumoniae* [35,85]. Furthermore, in our bacterial collection, we identified one *E*. *coli* isolate, one *K. oxytoca* isolate and numerous *K. pneumoniae* isolates with the hmv phenotype (unpublished data). In all these isolates, the *rmpA* and *rmpA2* genes were missing. This phenomenon questions the existence of another mechanism to express hypermucoviscosity [35,85]. Therefore, the mechanism by which these strains acquired this phenotype has yet to be determined. The hmv phenotype itself is considered a virulence trait [86]; however, in a nonhypervirulent background, its implication in human infection has been poorly characterized.

### Hypervirulent *K. variicola*

The hypervirulent (hv) phenotype was initially described in the *K. pneumoniae* ST23 clone. This emerging clone has been a threat to human health [87–89]. Until a few years ago, it was thought that hypervirulence was restricted to *K. pneumoniae*; however, years later, *K. quasipneumoniae* subsp. *similipneumoniae* and *K. quasipneumoniae* subsp. *quasipneumoniae* were found to acquire the hv phenotype by means of recombination events [90,91]. Hypervirulence associated with a multidrug-resistant phenotype has also been described, although it appears less frequently [54,79,92]. Nonetheless, the trend is toward the convergence of both phenotypes becoming a major challenge for clinical management [79,93–95]. We identified two reports of hypervirulent *K. variicola* in the literature (Table 1). The first report (R1) corresponds to an hvKv and colistin-resistant strain (WCHKV030666), recovered from blood cultures of a hospitalized Chinese patient [79]. Genomic analysis revealed the presence of a large plasmid, pVir_030666 (236 kb), in which multiple virulence genes commonly found in strains of hvKpn were identified. Among them, the plasmid-borne genes were the regulators of hmv phenotype *rmpA* and *rmpA2* and three siderophore systems, namely, aerobactin, salmochelin and yersiniabactin. Commonly, the yersiniabactin siderophore is chromosomally located [96]; however, in this case, the siderophore system was found on the virulence plasmid. The strain WCHKV030666 lacked the hmv phenotype owing to a frameshift mutation in the *rmpA*2 gene; however, the role of the *rpmA* gene in this genetic background is not clear. Interestingly, when the authors compared the sequence of the pVir_030666 plasmid against pLVPK [97], the well-studied virulence plasmid of hvKpn suggested that more virulence genes are carried by pVir_030666 due to differences in genetic structure and size. Like other virulence plasmids, pVir_030666 is not self-transmissible; this trait is associated with an incomplete conjugation system plus the presence of an *IS3* in the *traG* gene rather than the absence of the complete conjugation system, which is very common in other virulence plasmids [66].

The second report of hypervirulent *K*. *variicola* (R2) corresponded to a multidrug-resistant (MDR) clone causing a fatal outbreak in neonates with high mortality associated [56] (Table 1). Unlike the strain described in the work by Lu et al. (2018) [66], the outbreak clone possessed only classical virulence factors such as an iron acquisition system, fimbriae and urease utilization genes. The *K. variicola* clone lacked the *rmpA* and *rmpA2* genes, and the *in vivo* pathogenicity of *K. variicola* was tested in the *G. mellonella* model and compared to that of the hypervirulent strain of *K. pneumoniae* ST23 (A58300). The results suggested that *K. variicola* was more pathogenic than *K. pneumoniae* ST23. In this case, the hypervirulence may have been driven by new virulence determinants distinct from those reported in hvKpn strains. Moreover, the presence of the carbapenemase NDM-1 and the β-lactamase CTX-M-15 were identified, which is consistent with the convergence of multidrug resistance and hypervirulence [56]. In addition, the authors found inconsistencies when determining the capsular type, which suggests a novel KL locus with respect to the described *K. pneumoniae* serotypes.

In summary, this section has discussed *K. variicola* distinct phenotypes and highlights the importance of genomic evidence in order to establish associations between phenotypic traits and genomic traits. *K. variicola* mostly displays the classic phenotype, and although *K. variicola* is susceptible to most antibiotic classes, an increasing number of MDR strains have been reported. The virulence factors and the implications for the pathogenesis of *K. variicola* are not well studied. Thus, to understand the pathogenesis and virulence, studies that include not only associations between virulence factors but also STs and capsular types are needed. Some authors suggest that infections caused by cKv are as severe as those caused by cKpn*.* Since hvKpn variants were described much controversy regarding the hmv phenotype has been generated. It is known that hypermucoviscosity is not enough to define hypervirulent strains; however, the main role of this phenotype in strains that lack hypervirulent properties is not clear. Interestingly, hypervirulence in *K. variicola* may not be determined by the same traits as those present in hvKpn. As mentioned above, hvKv displays a genetic and genomic context distinct from that of hvKpn, and even more clinical parameters differ in hvKv. None of the *K. variicola* strains reported as hypervirulent exhibit the hypermucoviscous phenotype, but the only hypermucoviscous strain does not appear to be hypervirulent.

## MLST scheme for typing *K. variicola* isolates

The MLST is a powerful tool for epidemiological investigation of bacterial pathogens, allowing research on the genetic relatedness of bacterial isolates [98]. The MLST scheme for *K. pneumoniae* is used for typing isolates within the *K. pneumoniae* complex, such as *K. variicola* and *K. quasipneumoniae*. This section discusses the available MLST schemes and also addresses some pitfalls of using the *K. pneumoniae* MLST scheme and the advantages of having a specific *K. variicola* MLST scheme.

The *K. pneumoniae* MLST scheme works for *K. variicola*, and different studies mentioned in this review have applied the *K. pneumoniae* MLST scheme for typing *K. variicola* isolates [1,8,11,14,56]. Although a vast proportion of *K. variicola* genomes (54% 78/145) do not have an ST assignation or have a novel allele combination, previous authors have not discussed any possible reason why these isolates do not have a defined ST [11,14]. It is alarming that some important MDR clones are not assigned to an ST because this may limit molecular epidemiology of MDR clones among *K. variicola*. Moreover, Long et al. [11] suggest that the *K*. *pneumoniae* MLST typing scheme would misidentify *K. variicola* as *K. pneumoniae,* as the database contains information about *K. pneumoniae* isolates, which may contribute to the problems in identification.

*K. variicola,* like the other members of the *K. pneumoniae* complex, are distinct species [1,2]. For an epidemiological purpose, a specific MLST typing scheme may be needed to trace the genetic relationship and evolutionary history within *K. variicola* isolates. Recently, a research group developed a MLST typing scheme for *K. variicola* (http://mlstkv.insp.mx) [80]*.* This scheme is based on 7 housekeeping genes; one of them (*pyrG* gene) is a unique gene in *K. variicola*, which may promote the proper identification of *K. variicola*. This MLST uses the whole-genome sequence or the sequences of the seven housekeeping genes to determine the ST. In addition, when the whole genome sequence is used, the ANI has been implemented to identify which bacterial species of the *K. pneumoniae* complex are being submitted for ST assignment. In this case, it only assigns an ST for *K. variicola* genomes. This study included 254 genomes and isolates with a total of 166 distinct sequence types (STs). A global distribution for some STs was observed, and in some cases, isolates obtained from different sources belonged to the same ST. Interestingly, kingdom-crossing bacteria from plants to humans were identified, establishing this as a possible route of transmission of *K. variicola*. In addition, Clonal Complex 1 (CC1) was identified as the clone with the greatest distribution.

Additionally, the authors compared both typing schemes (*K. pneumoniae* and *K. variicola* MLST schemes) using *K. variicola* genomes. Interestingly, they found that by applying the *K. pneumoniae* MLST scheme in *K. variicola* isolates*,* it was difficult to establish genetic relationships. This was observed when isolates of *K. variicola* have a genetic relationship using *K. variicola* MSLT, and using the *K. pneumoniae* MLST, are completely dispersed. This is because the isolates of *K. variicola* are being related with isolates of *K. pneumoniae* than to those of their own species [80]. In summary, the comparison revealed that *K. variicola* isolates were dispersed among *K. pneumoniae* isolates, making it difficult to determine true relationships [80].

The unsuitability of standard techniques for distinguishing *K. variicola* from the rest of the *K. pneumoniae* complex has masked its epidemiological context. However, recent genomic and phylogenomic studies as well as molecular epidemiology analysis [80] (Table 1) have been conducted on *K. variicola* isolates. The authors consider the *K. variicola* MLST scheme is a strong molecular epidemiological tool that allows following the presence of MDR clones and the evolution of this bacterial species in different environmental niches [99].

## Genomic population structure

Recent insights into the population structure of *K. variicola* have revealed two genomic lineages (L1 and L2) that are not restricted by geographic area, host or antimicrobial resistance patterns. L2 is composed of the largest number of genomes (143/145) distributed among 26 clades and two distant genomes conforming to L1 [14]. Previous phylogenomic analyses have demonstrated that *K. variicola* is derived from a common ancestor and possesses a genome with a low frequency of recombination events [1,11]; however, recombination between *K. pneumoniae* complex members occurs and may be driven by the extensive genetic diversity within these species [11,14]. With the increasing number of new uploaded *K. variicola* genomes, differences in the population structure are likely to be observed as new lineages and subspecies.

## Concluding remarks and future perspectives

*K. variicola* is an important human pathogen, and over the years, it has been misidentified as *K. pneumoniae.* This misidentification has caused unawareness about significant aspects of this bacterial species. Great efforts have been made to differentiate *K. variicola* from the *K. pneumoniae* complex and to highlight the importance of this species as a cause of severe infections, such as BSIs and UTIs, two major HCaIs commonly attributed to *K. pneumoniae. K. variicola* is becoming a public health concern not only for the infections that it can cause but also due to its potential to acquire antimicrobial and virulence genes, complicating the clinical management of the infections produced by *K. variicola.* There are several methods to correctly identify *K. variicola*; however, they have not been routinely adopted, and as it currently stands, incorrect classification continues to be a problem. *K. variicola* possesses a particular set of virulence genes that are not completely determined; the investigation of new or additional virulence factors will contribute to depicting its virulome. Most of the typing schemes (capsule and MLST) are based on data obtained for *K. pneumoniae;* however, it would be feasible to design specific tools for characterizing *K. variicola* due to the distinctive characteristics of this species compared to those of the *K. pneumoniae* complex. In addition, the wide distribution of *K. variicola* in the environment and its potential use in industrial processes is clear evidence that the organism has its own molecular typing system. The implementation of methods that allow for proper differentiation of *K. variicola* from the *K. pneumoniae* complex should be implemented both at the clinical level and in experimental laboratories. Considering the different risks involved in facing an infection caused by *K.* variicola, correct identification of the microorganism that causes a particular infection will benefit patient outcomes. Regarding the information in the present review, we consider *K. variicola* to be an emerging pathogen in humans.
